# Osteopenia-osteoporosis discrimination in postmenopausal women by ^1^H NMR-based metabonomics

**DOI:** 10.1371/journal.pone.0217348

**Published:** 2019-05-29

**Authors:** T. A. Pontes, A. D. Barbosa, R. D. Silva, M. R. Melo-Junior, R. O. Silva

**Affiliations:** 1 Biology Applied to Health Postgraduate Program. LIKA–Laboratory of Immunopatology Keizo Asami. Universidade Federal de Pernambuco, Av Prof Luis Freire, s/n. Cidade Universitaria, Recife-PE, Brazil; 2 Fundamental Chemistry Department, CCEN. Chemistry Postgraduate Program. Universidade Federal de Pernambuco. Av. Jornalista Aníbal Fernandes, s/n. Cidade Universitária, Recife-PE, Brazil; Instituto de Investigacion Sanitaria INCLIVA, SPAIN

## Abstract

This is a report on how ^1^H NMR-based metabonomics was employed to discriminate osteopenia from osteoporosis in postmenopausal women, identifying the main metabolites associated to the separation between the groups. The Assays were performed using seventy-eight samples, being twenty-eight healthy volunteers, twenty-six osteopenia patients and twenty-four osteoporosis patients. PCA, LDA, PLS-DA and OPLS-DA formalisms were used. PCA discriminated the samples from healthy volunteers from diseased patient samples. Osteopenia-osteoporosis discrimination was only obtained using Analysis Discriminants formalisms, as LDA, PLS-DA and OPLS-DA. The metabonomics model using LDA formalism presented 88.0% accuracy, 88.5% specificity and 88.0% sensitivity. Cross-Validation, however, presented some problems as the accuracy of modeling decreased. LOOCV resulted in 78.0% accuracy. The OPLS-DA based model was better: R2Y and Q2 values equal to 0.871 (p<0.001) and 0.415 (p<0.001). LDA and OPLS-DA indicated the important spectral regions for discrimination, making possible to assign the metabolites involved in the skeletal system homeostasis, as follows: VLDL, LDL, leucine, isoleucine, allantoin, taurine and unsaturated lipids. These results indicate that ^1^H NMR-based metabonomics can be used as a diagnosis tool to discriminate osteoporosis from osteopenia using a single serum sample.

## Introduction

Osteoporosis is a multifactorial systemic skeletal disease that causes damage to the microarchitecture of bone tissue, increasing the risk of fractures [1]. Women in the postmenopausal period are the most affected by this problem because of the hormonal deficiency that occurs during this period. Reduction of estrogen levels promotes the homeostatic imbalance of the bone remodeling process, causing an increase in bone resorption, deterioration of the microarachitecture, and a decrease in bone mass. About 40% of women older than 50 years of age are diagnosed with postmenopausal osteoporosis, making it necessary to pay special attention to this patient group [2]. Estrogen hormone therapy has been considered the most effective for the prevention and treatment of postmenopausal osteoporosis. However, investigations showed that estrogen could lead to higher occurrences of endometrial cancer, stroke, cardiovascular diseases and breast carcinoma [3]

According to WHO criteria, the osteoporosis diagnosis is performed from bone mineral density (BMD) determination, using a *T*-score. Patients with bone mineral density values (in *T*-score) higher than –1.0 are classified as healthy, while patients with *T*-score minor than –2.5 are diagnosed with osteoporosis. Patients who have *T*-scores between –2.5 and –1.0 are not classified as having osteoporosis, but also present risk of fractures higher than the medium of the population. These patients are diagnosed with osteopenia [4].

The pharmacological approach is generally recommended for patients with osteoporosis and osteopenia, as indicated by the fracture risk assessment (FRAX), which takes into account other factors (BMI, used medications, previous fracture, etc.) in addition to BMD. However, the decision of pharmacological treatment carries with it the risk of adverse effects and side effects. Thus, early diagnosis of bone loss allows an intervention only with changes in patient lifestyle [5,6].

Some serum biomarkers of bone formation (or resorption) are used to this diagnosis. As the resorption process is faster than bone formation, levels of bone resorption biomarkers change more rapidly than levels of bone formation markers [7–9]. In addition, others metabolites are studied with correlation in bone metabolism. Tanko et al.[10], Orozco et al.[11] and Poiana et al.[12] have reported that postmenopausal women who presented abnormal lipid profile had lower lumbar and femoral BMD and, therefore, higher risk of fractures. They suggest an association between hyperlipidemia and osteopenia. Moreover, amine-terminal collagen type I (NTX-I) telopeptides, which are markers of bone resorption, present positive correlation with Total Cholesterol and LDL serum levels [13]. Lv et al.[14] identified changes in arachidonic acid, leucine, isoleucine, lactate, taurine and cholesterol serum levels and showed that these changes correlated with loss of bone mass.

Metabonomics applies multivariate statistical formalisms to spectral data aiming to correlate them to biochemical status. In metabonomics strategy, the working hypothesis is that when life system is exposed to an external agent, by homeostasis, there are changes in the concentration of endogenous metabolites which can be associated to patients’ biochemical status. Therefore, it is possible develop metabonomics models aiming to diagnose or assess a clinical therapy [15,16]. There are various reports that make use of metabonomics for clinical diagnosis. Duarte and Gil [17] showed the use of metabonomics in the study of human biofluids to identify various types of cancer, using ^1^H NMR spectra. Qiao et al.[18] studied schizophrenia in patients treated with olanzapine through the metabolic analysis of blood plasma. Gouveia et al.[19] developed metabonomics models to investigate periportal liver fibrosis caused by *mansonic schistosomiasis* among patients diagnosed with viral hepatitis. Batista et al. [20] discriminated intermediate from advanced liver fibrosis in patients using ^1^H NMR-based metabonomics. Godoy et al.[21] used LDA metabonomics model to made hepatitis C virus diagnosis from urine ^1^H NMR spectra.

Statistical formalisms employed in metabonomics assays are divided in two categories: unsupervised, which doesn’t use class information, and supervised methods. Principal Components Analysis (PCA) is the unsupervised method more used to investigate natural grouping and outlies. The samples and original variables are projected in new coordinate system defined by Principal Components (PC), producing case and loading plots, respectively. Each PC explains part of variance contained in dataset. Among unsupervised methods, Discriminants Analysis formalisms are the more employed, with highlight to PLS-DA (Partial Least Discriminants Analysis), OPLS-DA (Orthogonal Partial Least Discriminants Analysis) and LDA (Linear Discriminants Analysis). The discriminants analysis formalisms are linear combinations of original variables that relate to class matrix (matrix Y). In PLS-DA are build Latent Variables, similar to PCA, but considering the variance contained into matrix Y; in OPLS-DA, the systematic variance contained in matrix X (dataset) is divided in two groups, where first component explains the higher variance contained into matrix Y associated to difference between the classes, while the second group, called orthogonal component, explains intraclass variance; LDA is a linear combination of the some original variables. Therefore, LDA needs to use a variable selection tool to build the discriminant function which will divide to space in two regions, where the samples of each class are projected [15,16,22].

Our study was to use metabonomics strategy to discriminate osteopenia from osteoporosis in postmenopausal women, using ^1^H NMR spectra of serum.

## Materials and method

### Patients and ethical procedure

The study was developed using samples of postmenopausal women arising from Cabo de Santo Agostinho city (Pernambuco/Brazil). These patients were recruited by spontaneous demand when they were to Rheumatology Ambulatory, where were submitted to anamnesis and the bone mineral density (T-scores) was determined using the Hologic Bone Densitometer Discovery Ci. For each patient, T-scores were measured in three regions–lumbar spine (L1-L4), femoral neck and femur total, being considered the site with minor T-score. Bone mineral density assays were performed until 90 days before ^1^H NMR analysis. The body mass index (BMI) of each patient and serum level of total cholesterol and alkaline phosphatase were determined. In the study were excluded patients with others associated chronic disease as well as those who were making use of drugs that affect BMD. After anamnesis, were recruited 78 volunteers who were distributed in three groups: (1) Healthy, containing twenty-eight women; (2) Osteopenia, being twenty-six patients; and (3) Osteoporosis, containing twenty-four patients. This study received approval from the Ethics Committee of the Universidade Federal de Pernambuco Health Sciences Center (Approval number 1.114.754/July 2015) and all volunteers signed the Free and Informed Consent Term.

### Statistical analysis

The clinical parameters of participants (mean age, bone density, body mass index, total cholesterol and alkaline phosphatase) were submitted to statistical analysis through ANOVA and Tukey test with significance level of 5% (p > 0.05), using the GraphPad Prism version 7.0 (GraphPad Software Inc., USA).

### Metabonomics assay

^1^H NMR spectra were performed using a VNMRS400 spectrometer operating at 400 MHz. Samples were prepared using 400 μL of serum and 200 μL of D_2_O. Acquisition used the following parameters: T2-filter associated to presaturation of water signal (Presat-CPMG) pulse sequence, as follows: spectral window equal to 6.4 kHz, acquisition time equal to 2.56 s, 128 transients, spin echo delay equal to 400 μs, 88 cycles, giving a total echo time equal to 70.4 ms and saturation delay equal to 2.0 s. [23] The signal attributed to methyl group of lactate (δ 1.33 ppm) was used as chemical shift reference. Spectra were binned in the region between δ 8.00 and 0.00 ppm, 0.04 ppm/bin, excluding the region between δ 5.12 and 4.48 ppm. Spectral data were collected in a matrix with 78 cases (lines) and 184 variables (column). Data set were preprocessed using normalization by sum (in line) and employed PCA, PLS-DA and OPLS-DA formalisms, using MetaboAnalyst online platform. Metabonomics models were validated using Leaving-One-Out Cross Validation (LOOCV) and permutation test, using 2000 permutations. LDA Model was performed using Statistical 10.0 software. The selection of variables to build LDA model was performed using Wilk’s Lambda.

## Results

**Fig 1:**
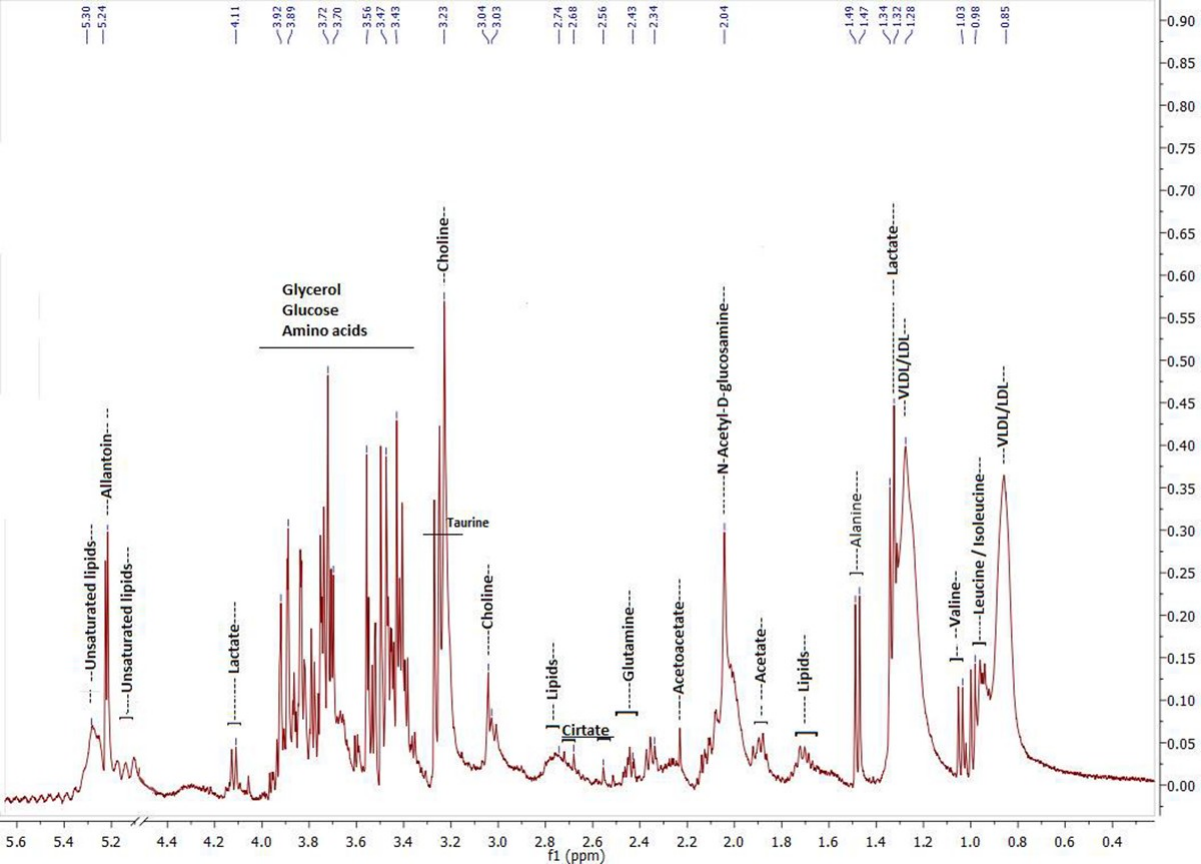
Typical ^1^H NMR spectrum of serum (400 MHz, D_2_O, Presat-CPMG pulse sequence) used in the study.

Table 1 shows clinical data of participants of each group, while Fig 1 presents a typical ^1^H NMR spectrum of serum obtained in this study and main assignments, identifying associated metabolites.

**Table 1:** Clinical data of studied volunteers.

		Studied Groups				
		Healthy volunteers (1)	Osteopenia patients (2)	Osteoporosis patients (3)	ANOVA p-value^b^	Tukey test p-value^c^
Bone Density (*T*-scores)^a^		-0.11 (±0.7)	-1.64 (±0.4)	-3.11 (±0.5)	<0.0001	<0.0001^d^
BMI (kg.m^-2^)		25.35 (±3.4)	27.20 (±5.2)	25.58 (±4.8)	0.2165	-
Cholesterol Total (mg.L^-1^)		216.28 (±28.2)	226.4 (±37.3)	217.7 (±40.8)	0.503	-
Alkaline Phosphatase (mg.L^-1^)		78.0 (±36.9)	75.4 (±40.7)	102.5 (±58.1)	0.3351	-
Age (years old)		60.38 (±6.2)	61.88 (±7.9)	60.80 (±6.0)	0.5292	-
Ethnnicity	Afrodescendant	20 [71%]	17 [65%]	16 [67%]	0.9916	-
	Caucasian	7 [25%]	8 [31%]	6 [25%]		
	Not declared	1 [4%]	1 [4%]	2 [8%]		
N		28	26	24		-

^a^*T*-scores were measured in three regions–lumbar spine (L1-L4), femoral neck and femur total It was considered the site with minor value. BMD collected until 90 days before ^1^H NMR analysis.

^b^ Fisher’s chi-square test.

^c^ Only when p-value of ANOVA < 0.05

^d^ It was observe the same p-value when compared all studied groups: (1) *vs* (2); (1) *vs* (3); and (2) *vs* (3).

**Fig 2:**
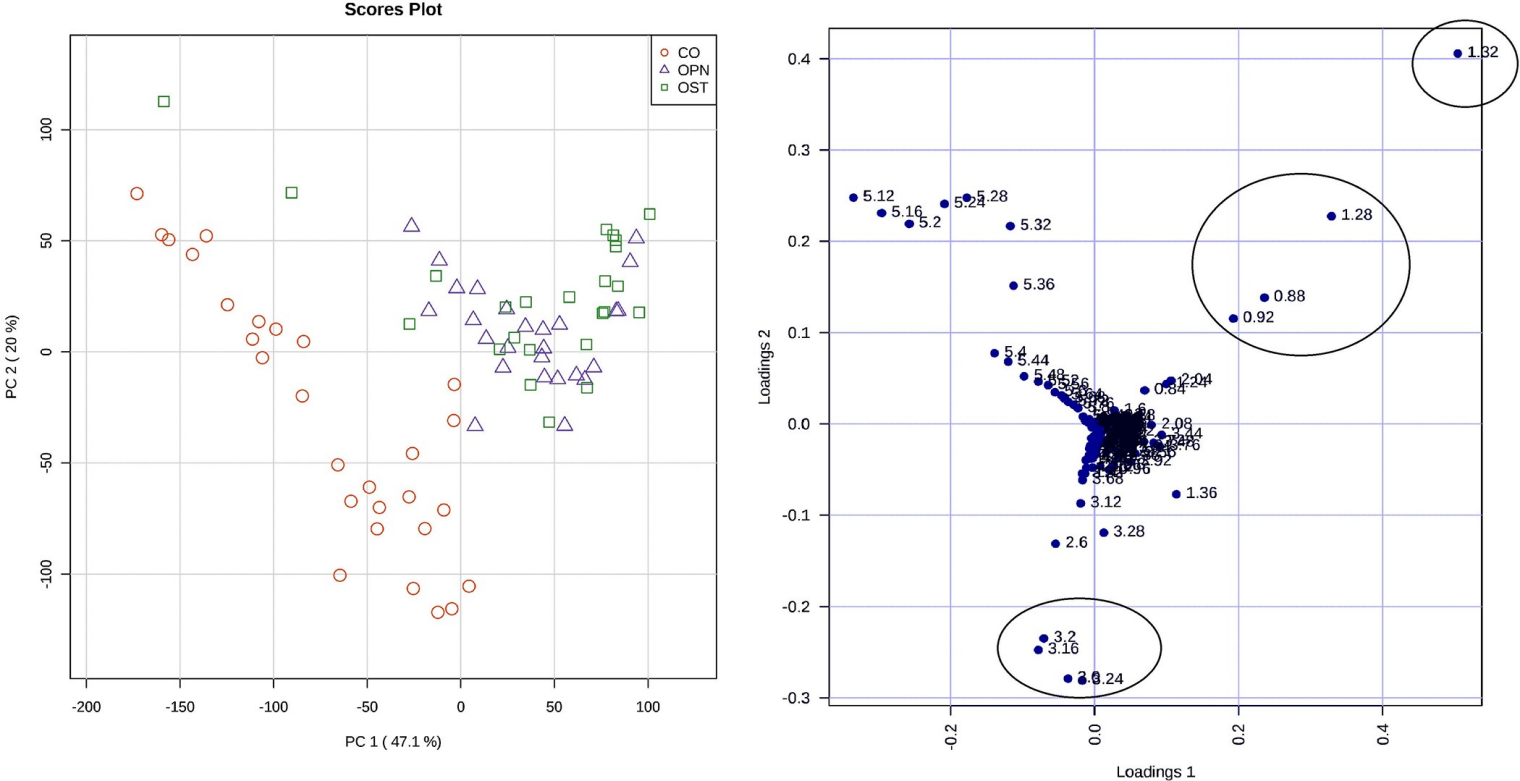
PCA results using all dataset. On the left, score plot (PC1xPC2, 71% of variance)—control (red circles), osteoporosis (green squares) and osteopenia (blue triangles); The right, loading plot indicates the most important variables for discrimination.

The study used serum samples from 78 volunteers. Exploratory analysis was performed using PCA formalism and results are presented in Fig 2. PC1 and PC2 explain 71% of variance contained in the dataset. Separation between healthy volunteers and patients (osteopenia or osteoporosis) samples can be observed. There is no natural discrimination, however, between the osteopenia and osteoporosis groups. The PCA loading plot indicates that regions δ 0.88–1.32 ppm and δ 3.12–3.28 ppm are important to discriminate diseased from healthy volunteers. The control group presented higher intensity to signal at δ 3.12–3.28 ppm, while diseased volunteers presented a more intense signal at δ 0.88–1.32 ppm.

When PCA formalism was applied to the osteopenia/osteoporosis data, neither separation was observed. Then, supervised methods were employed, such as LDA (Linear Discriminant Analysis) and OPLS-DA (Orthogonal Partial Least Square–Discriminant Analysis), aiming to discriminate between the osteopenia and osteoporosis samples. A LDA model was built using six variables, as follows: δ 0.92, 1.28, 3.24, 3.68, 5.44 and 5.96 ppm. Table 2 shows the classification matrix using the LDA model.

**Table 2:** Samples classification based on scores obtained from LDA Model using fifty ^1^H NMR spectra– 26 osteopenia and 24 osteoporosis.

		Clinical Diagnosis	
LDA Model		Osteopenia	Osteoporosis
	Osteopenia	23 (21)*	3 (5)*
	Osteoporosis	3 (6)*	21 (18)*

*F*_(6,43)_ = 8.60 p<0.001

*Classification after LOOCV.

The LDA model presented sensitivity, specificity and accuracy values equal to 88.0%, 88.5% and 88.0%, respectively. The *F*-test with 6 (variables used) and 43 (50-6-1) degrees of freedom was equal to 8.60 (p<0.001). After LOOCV, LDA model presented 78.0% accuracy, 75% specificity and 80.8% sensitivity.

**Fig 3:**
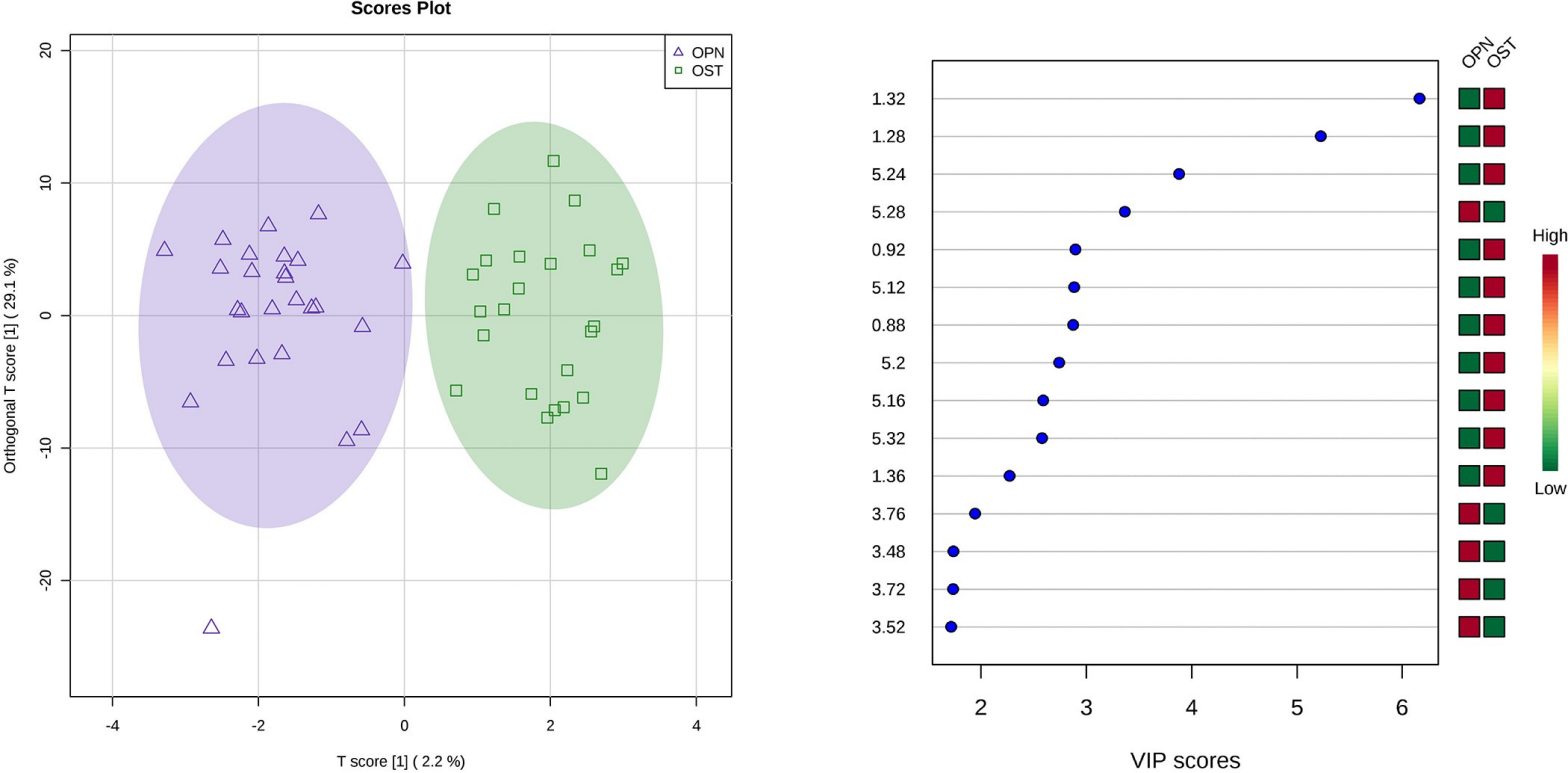
OPLS-DA results using only osteoporosis (green squares) and osteopenia (blue triangles) samples from postmenopausal women. Score plot (at left) and VIP score plot (at right).

Fig 3 presents results obtained applying OPLS-DA formalism to the osteopenia-osteoporosis dataset (fifty samples). OPLS-DA model presented R2Y and Q2 values equal to 0.871 (p<0.001) and 0.415 (p<0.001), respectively, after validation by permutation test.

The VIP scores plot (Fig 3, right) indicates some spectral regions which are responsible for discrimination, as follows: δ 3.48–3.76 ppm and δ 5.28 ppm, where the signal intensities are higher in the osteopenia group than in the osteoporosis group; δ 0.88–0.92 ppm; 1.28–1.36 ppm; and 5.12–5.32 ppm, where the signals are more intense in osteoporosis group.

## Discussion

International Osteoporosis Foundation [24] data indicate that osteoporosis was diagnosed in more de 200 million women and is associated with 9 million fractures annually in the world. Generally, osteoporosis is associated to women and to aging, but can be diagnosed in the young and also in men. About 33% of women over 45 years old have a positive diagnosis for osteoporosis. When women over 80 are observed, the disease reaches about 73% of this population. This indicates that is important to develop diagnostic tools that are able to discriminate osteoporosis from osteopenia patients in this group (postmenopausal women). The present study investigated only postmenopausal women, aiming to discriminate osteopenia from osteoporosis, using ^1^H NMR-based metabonomics. There were three groups–Healthy (28 volunteers), osteopenia (26 patients) and osteoporosis (24 patients).

Table 1 indicates that there is only significant difference in bone mineral density. This is natural, since bone mineral density is the common criteria to diagnose a patient. The groups were similar in all the other parameters studied. The PCA loading plot (Fig 2) suggests that the lipid profile of patients was slightly different from healthy volunteers, since that variables δ 0.88–1.32 ppm are important to discriminate diseased from healthy samples. The Control group presented greater signal intensity at δ 3.12–3.28 ppm, while osteopenia/osteoporosis patients presented increased integration values at δ 0.88–1.32 ppm. Signals at δ 3.12–3.28 ppm were attributed to choline and taurine, while signals at δ 0.88–1.32 ppm were attributed to methylene and methyl groups of VLDL and LDL. These results are in according with reports that associated decrease in bone mineral density to increased LDL serum level [14,25]. PCA loading plot shows that the control group samples signals were more intense at δ 3.16–3.24 ppm, indicating that healthy volunteers had choline and taurine serum levels higher than the osteopenia/osteoporosis patients. This was also reported by Long et al.[26] and Lv et al.[20], who observed a decrease in choline concentration in patients diagnosed with osteoporosis. Taurine is one of the most abundant non-essential amino acids found in bones. This result corroborates with studies correlating taurine as an osteoclast formation inhibitor and osteoblast inductor [2,27,28]. Besides that, choline and tyrosine amino acids were identified as important for the discrimination between healthy volunteers and osteopenia-osteoporosis patients, with higher concentrations observed in the control group. This observation agrees with reports in the literature [14,26], [29,30] which indicate that choline and tyrosine concentrations decreased in patient with osteoporosis. Tyrosine is one of the amino acids present in thyroid hormones (T3 e T4) with an intimate relationship with the osseous metabolism and stimulates the expression of genes in the osteoblasts for the production of collagen [29,30].

Exploratory analysis, however, did not discriminate osteoporosis from osteopenia. Supervised formalisms were employed aiming at this discrimination. A LDA Model was built using six variables which were associated with leucine, isoleucine, lactate, taurine and unsaturated compounds. This metabonomics model is significant statistically, since the *F*-test with 6 and 43 degrees of freedom was equal to 8.60 (p<0.001). However, there was a grey area in the model, where the classification was doubtful. This was evidenced when LOOCV (Leave-One-Out Cross Validation) was carried out, resulting in a 78.0% accuracy. Alternatively, OPLS-DA was used and the metabonomics model built was able to discriminate between the groups, indicating that there was a significant difference between them. The validation using 2000 permutation resulted in R2Y and Q2 values equal to 0.871 (p<0.001) and 0.415 (p<0.001), respectively. Fig 3 shows four spectral regions important for discrimination: δ 0.88–0.92 ppm; δ 1.28–1.36 ppm; δ 3.48–3.76 ppm; and δ 5.12–5.32 ppm.

According to the OPLS-DA VIP score (Fig 3), there was an increase in the allantoin serum level in the osteopenia group while the osteoporosis groups presented higher serum levels of cholesterol, lactate and unsaturated lipids. These findings already have been reported by Chen et al.[31], who observed an increase in allantoin serum level after osteoporosis prophylaxis. Maritz et al. [25] and Lv et al.[20] reported an association between cholesterol serum level and osteoporosis diagnosis. Xue et al.[3] and Dixon and Sims[32] associated lactate serum level with osteoblasts inhibition and osteoclasts formation. While Xue et al.[3], Lv et al.[20] and Parhami et al.[33] all reported that the products of lipids oxidation are associated to osteoblast differentiation inhibition. Table 3 summarizes the metabolites that are associated to observed discriminations in this study, as well as indicates data from the literature that show the relation among these metabolites and the diagnosis of osteoporosis.

**Table 3:** Identification of metabolites in the metabonomics model responsible for discrimination among groups.

Compound	nuclei and (δ/ppm)	↑ group	References
Cholesterol (VLDL/LDL)	CH_3_ (0.88 ppm) CH_2_ (1.28 ppm)	OST	[20], [25]
Leucine and isoleucine	γ-CH_3_ (0.92 ppm)	OST	[34]
Lactate	CH_3_ (1.32 ppm)	OST	[3], [32]
Tyrosine	β-CH_2_ (3.16 ppm)	Control	[30], [29]
Choline	CH_3_N (3.20 ppm)	Control	[26] [20]
Taurine	CH_2_N (3.24 ppm)	Control	[2,27,28]
Allantoin	CH (5.28 ppm)	OPN	[31].
Unsaturated lipids	= CH (5.15–5.28 ppm)	OST	[33],.[3], [20].

OPN—Osteopenia; OST—Osteoporosis; ↑ higher concentration

Therefore, all formalisms employed indicated that it is possible to discriminate osteopenia from osteoporosis using serum ^1^H NMR spectra of patients. The best metabonomics model was built using OPLS-DA formalism which also revealed the metabolites associated to discrimination. These findings are important for clinical practice, since that is nothing change in the routine of patients is employed; the assay is minimally invasive; it is not necessary the presence of patient neither doctor during the analysis, contributing for decrease the length of stay of patients in the hospital environment and decreasing the probability of infections, for example. However, the main gain associated to introduction of ^1^H NMR-based metabonomics in the clinical practice will be to obtain patients’ systemic information in the first exams requested by doctors. Besides of metabonomics models to differential diagnosis of osteopenia-osteoporosis, others various metabonomics models can be built for disease different helping in the early diagnosis.

### Conclusion

In this paper, three multivariate statistical tools were employed in serum ^1^H NMR spectra data aiming at an osteopenia-osteoporosis differential diagnosis. Principal Component Analysis discriminated healthy volunteers from osteopenia-osteoporosis patients, but didn’t discriminate osteopenia from osteoporosis. This differentiation was obtained only when supervised methods were used–Linear Discriminant Analysis and Orthogonal Partial Least Square-Discriminant Analysis. The best result was obtained using OPLS-DA which presented R2Y and Q2 values equal to 0.871 (p<0.001) and 0.415 (p<0.001), respectively. Moreover, the metabonomics strategy used identified the metabolites associated with the discrimination observed. This permits us to understand the disease evolution mechanism and make a rapid and early differential diagnosis of osteopenia and osteoporosis in postmenopausal women.
